# Laser Plasma Jet Driven Microparticles for DNA/Drug Delivery

**DOI:** 10.1371/journal.pone.0050823

**Published:** 2012-11-30

**Authors:** Viren Menezes, Yohan Mathew, Kazuyoshi Takayama, Akira Kanno, Hamid Hosseini

**Affiliations:** 1 Department of Aerospace Engineering, Indian Institute of Technology Bombay, Powai, Mumbai, India; 2 Institute of Fluid Science, Tohoku University, Sendai, Japan; 3 Graduate School of Life Sciences, Tohoku University, Sendai, Japan; 4 Bioelectrics Research Center, Kumamoto University, Kumamoto, Japan; Universidad Nacional Autonoma de Mexico, Instituto de Biotecnologia, Mexico

## Abstract

This paper describes a microparticle delivery device that generates a plasma jet through laser ablation of a thin metal foil and uses the jet to accomplish particle delivery into soft living targets for transferring biological agents. Pure gold microparticles of 1 µm size were coated with a plasmid DNA, *pIG121Hm*, and were deposited as a thin layer on one surface of an aluminum foil. The laser (Nd:YAG, 1064 nm wavelength) ablation of the foil generated a plasma jet that carried the DNA coated particles into the living onion cells. The particles could effectively penetrate the target cells and disseminate the DNA, effecting the transfection of the cells. Generation of the plasma jet on laser ablation of the foil and its role as a carrier of microparticles was visualized using a high-speed video camera, Shimadzu HPV-1, at a frame rate of 500 kfps (2 µs interframe interval) in a shadowgraph optical set-up. The particle speed could be measured from the visualized images, which was about 770 m/s initially, increased to a magnitude of 1320 m/s, and after a quasi-steady state over a distance of 10 mm with an average magnitude of 1100 m/s, started declining, which typically is the trend of a high-speed, pulsed, compressible jet. Aluminum launch pad (for the particles) was used in the present study to make the procedure cost-effective, whereas the guided, biocompatible launch pads made of gold, silver or titanium can be used in the device during the actual clinical operations. The particle delivery device has a potential to have a miniature form and can be an effective, hand-held drug/DNA delivery device for biological applications.

## Introduction

The biolistic process that was first carried into effect as a transfection method by Klein et al. [1] was eventually proved to be an efficient method for DNA/drug delivery [2], [3] with minimal invasion on the target cells. In this method, the microparticles coated with biological agents were propelled at high speeds using suitable delivery devices to penetrate the intact living cells and disseminate the coated biological agents efficiently. As the effectiveness of this treatment method depended mainly on the penetration of the drug-coated microparticles to an appropriate depth in the target cells, the devices used for this purpose were to be appropriately modulated to impart the right momentum to the particles. Hence, a component of research was directed towards the development of reliable biolistic devices.

The popular biolistic devices developed in the past were essentially pneumatic [3], [4] or were driven collectively by pneumatic and inertial mechanisms [5]. The operation of the pneumatic devices was based on the theme of a shock tube and/or a wind tunnel, in miniature forms, where a high-speed (inert) gas in a tube carried the drug-coated microparticles onto the target surface. These devices were used for vaccine delivery into epidermis of living mice [3], [6] and DNA delivery into brain cells of living lampreys [4], and their pharmaceutical applications were well realized. But, a substantial mass of the high-pressure carrier-gas and the associated gas-boundary-layer effects in the device spacers raise apprehensions of their use on internal organs in human body. On the other hand, the pneumatic-inertial devices deployed a macro-projectile, the front surface of which contained drug-coated microparticles. The macro-projectile was driven at a high speed by a pneumatic mechanism (essentially pressurized gas) and was abruptly stopped by a stopper, thereby ejecting the microparticles out of its surface by inertia. The ejection of the microparticles and the placement of the biological target in this case take place in a chamber of coarse vacuum in order to reduce drag on the particles and effect their penetration to a desired depth in the target. Owing to the high-speed movement of a solid component in the device and the requirement of coarse vacuum to realize particle penetration into the target, the pneumatic-inertial devices could not turn out to be handy and were not proposed for medical/surgical procedures, and their use was limited to dissociated, cultured targets.

Biolistic delivery of drug into soft, internal targets in human body would require a device that is handy, well controlled and maneuverable. A device which can be miniaturized and integrated with minimally invasive surgical equipment such as endoscopes will be advantageous. The devices that use gas as the driver would require seamless conduits for an effective operation, which restrict their flexibility/accessibility and hence their adaptability to surgical equipment like endoscopes. In this backdrop a laser ablation assisted biolistic device was developed [7] and tested [8] on soft targets to check the viability of particle penetration and drug dissemination, leading to transfection of cells. While the device could effectively transfer particles to a sufficient depth for cell transformation in soft targets, its effectiveness on slightly hard targets was expected to be faint as the drug-coated microparticles underwent an extreme deceleration on leaving the launch pad [9]. The device accelerated the drug particles solely due to inertia, where the particles could not retain their momentum for long and decelerated quickly due to the atmospheric drag. The deceleration reduced the impact velocity of the particles at the target surface and limited the scope of their penetration into the target. In order to accelerate the particles further on leaving the launch pad, we thought of a continuous carrier for these particles and modulated the biolistic device to emanate a plasma jet from the rear of the launch pad. The emanant plasma jet prevented the microparticles from getting decelerated on leaving the launch pad and maintained a high impact velocity at the target surface, increasing the number of penetrations. This mode of operation of the device is believed to be useful in targeting harder living targets/cells in a biolistic process.

This paper presents the second mode of operation of the biolistic device reported by Nakada et al. [8]. The second mode of operation used a high-speed plasma jet to carry the accelerated drug particles onto the target. The specialty of this operation was that the particles did not undergo a severe deceleration as they had a carrier (plasma jet), which enabled them to sustain a high velocity till they reached the target surface. This feature of the device realized a better penetration and distribution of the particles in the living target. The particle cloud in the present case was observed to be accelerating after the launch, as visualized using a high-speed video camera, a characteristic that was expected to come about on introducing a particle carrier into the process. The present device can effect the particle delivery into harder targets.

The present biolistic device is laser based, where the laser beam makes it functional. The laser beam can be drawn either through an optical fiber or a miniature optical arm without any functional loss and the entire set-up can be diminished into a flexible, hand-held tool and can even be integrated with non-invasive surgical/medical devices like endoscopes to reach non-approachable treatment sites during a medical operation.

## Materials and Methods

### A. The Device and its Physics

**Figure 1:**
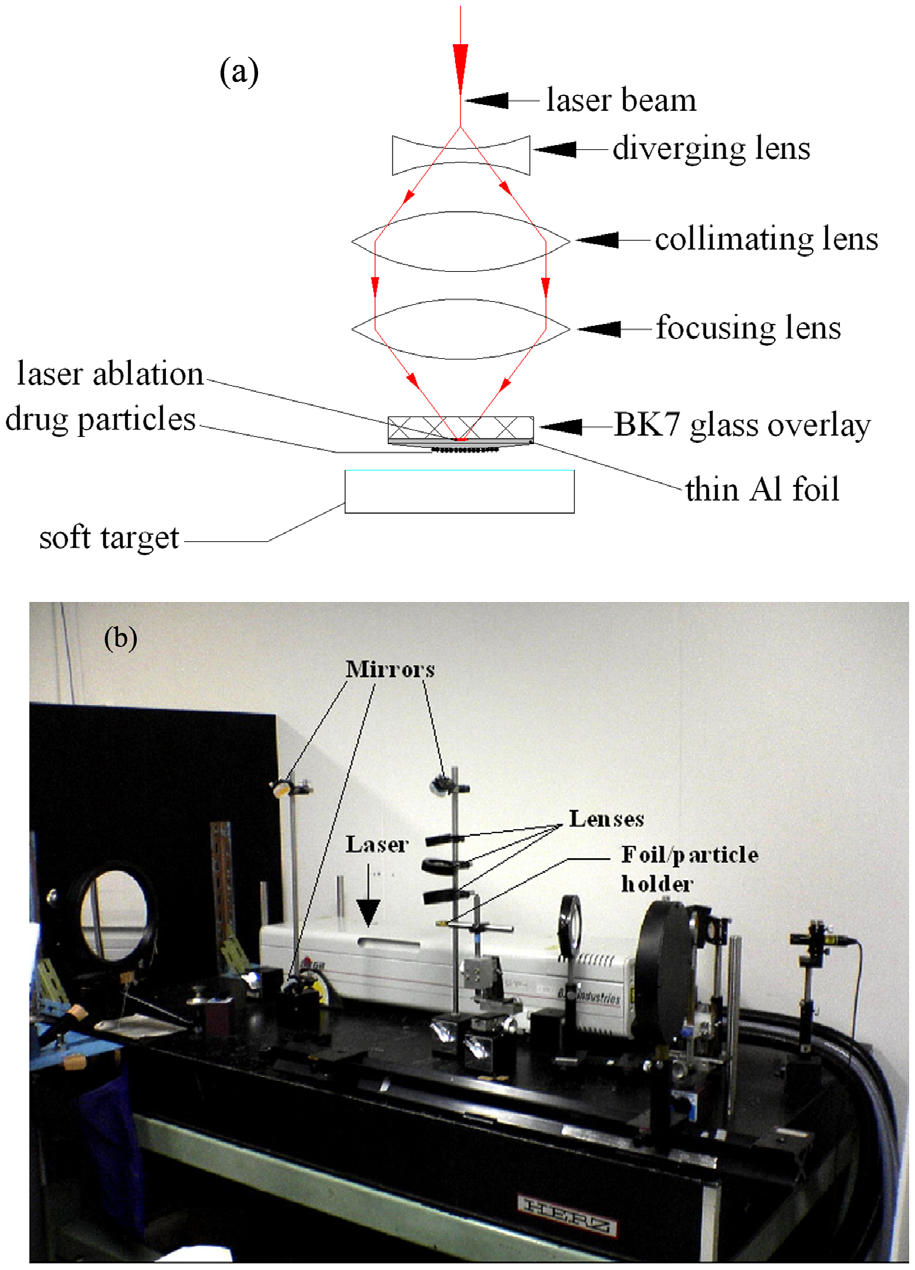
Organization of the experimental components. (a) Schematic of the device. (b) Photograph of the experimental set-up.

**Figure 2:**
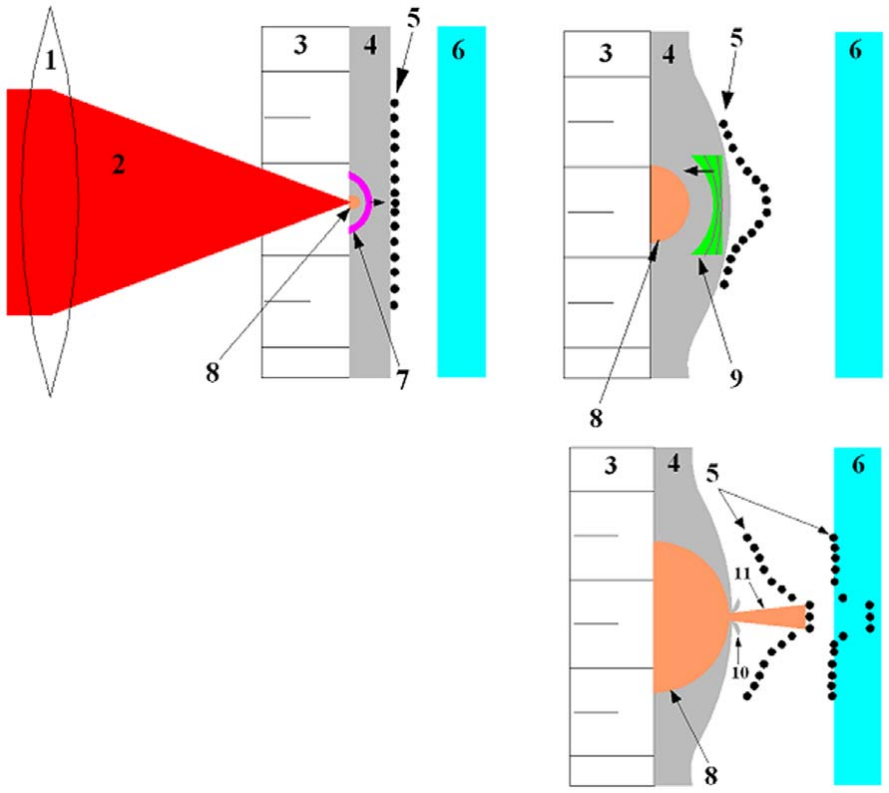
Schematic of the process of particle acceleration on laser ablation of a thin *Al* foil. [1− Lens. 2− Laser beam. 3− Glass overlay. 4− Foil. 5− Particles. 6− Target. 7− Shock wave. 8− Confined ablation. 9− Expansion wave. 10− Foil rupture. 11− Foil vapor/plasma jet].

The device/experimental set-up comprised an Nd:Yttrium Aluminum Garnet Laser (1064 nm wavelength, 5.5 ns pulse duration, 1.4 J/pulse energy) equipped with a suitable optical set-up (lenses and mirrors) for focusing, a BK 7 glass overlay of 20 mm diameter and 5 mm thickness, a thin, rectangular metal foil (typically 100 µm thick) made of either pure Aluminum (*Al*) or Gold (*Au*), and a circular metallic holder for housing the BK7 glass and the thin foil. The DNA coated gold particles of 1 µm size were deposited on the posterior surface of the foil as a thin layer. The anterior (front) surface of the foil was ablated using the laser beam over a spot of about 2 mm diameter with the maximum laser energy available (1.4 J/pulse). The laser ablation of the foil was confined by the BK7 glass overlay to enhance the ablation effects. The ablation caused the foil (on the ablation spot, to a part of its depth) to evaporate into ionized vapor, the sudden blow-off of which launched a shock wave through the foil and also ruptured the foil through an opening of a size of a millimeter, due to heat and pressure. Due to shock wave loading, the microparticles deposited on the posterior surface of the foil accelerated, and when the foil ruptured, the ionized vapor from the anterior side of the foil rushed out as a tiny jet, further carrying the already accelerated particles onto the targets placed in the vicinity of the foil. The schematic of the device, showing various parts, is presented in Fig. 1a. A photograph of the experimental set-up arranged on an optical bench is shown in Fig. 1b. The physical process of particle acceleration, as described above is depicted in Fig. 2.

### B. Biological Materials

The plasmid DNA, *pIG121Hm*, which contained the *nptII* (neomycin phosphotransferaseII) gene under the control of the *nos* promoter, the *hpt* (hygromycin phosphotransferase) gene under the control of the *CaMV* (cauliflower mosaic virus) 35*S* promoter, and the β-glucuronidase (GUS) gene with an intron (GUS-intron) under the control of the *CaMV* 35*S* promoter, was used [8]. The closed circular form of the plasmid DNA was purified and coated onto gold particles (Bio-Rad) of 1 µm size by co-precipitation in ethanol at a DNA concentration of 15 µg of DNA/mg of particles.

The biological targets used were the scales of onion (*Allium cepa*) that were cut into 1×1 cm^2^. The onion was purchased locally in Sendai, Japan.

## Results and Discussion

**Figure 3:**
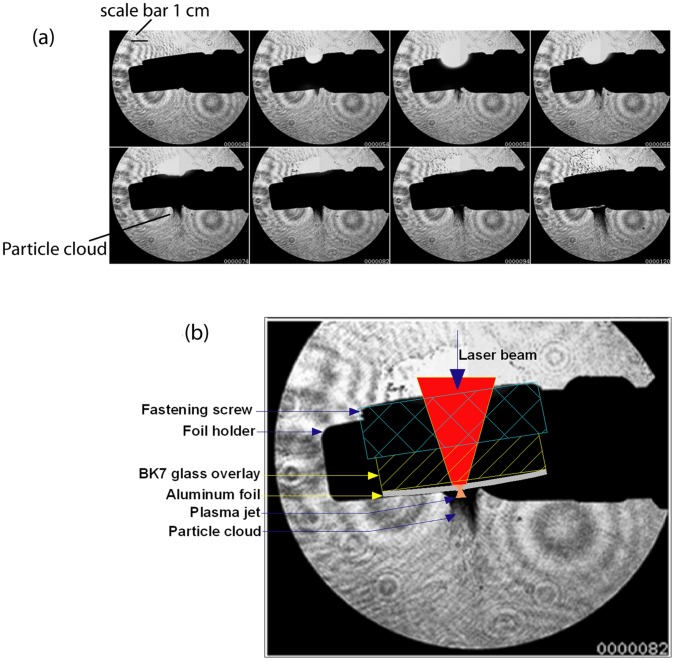
Photographs of microparticle acceleration on laser ablation of the foil/launch pad. (a) Sequent pictures of particle acceleration during an experiment. Particles are accelerating into atmospheric air (at 1 bar pressure). (b) Annotated frame elucidating the photographs in part (a).

The operation of the device was analysed through photography of the particle acceleration process using a high-speed video camera (Shimadzu HyperVision−HPV 1) aligned with a standard Shadowgraph. The photography was carried out at a sampling rate of 500-kilo frames per second. For the analysis of microparticle acceleration, tungsten particles of 1 µm size were used, as the tungsten particles have the same density as that of gold particles, and the particle dynamics did not change, while the cost of the test was reduced substantially due to the use of tungsten particles instead of gold. About 500 µg of tungsten particles (Bio-Rad) were deposited on the posterior surface of a 100 µm thick aluminum (99.2% purity, Nilaco Corporation) foil and the anterior surface of the foil was ablated using the laser beam as explained above. Figure 3a shows the photographs of the launch of tungsten microparticles from the foil surface on laser ablation. A photograph of the launch is magnified and annotated in Fig. 3b to illustrate the process.

**Figure 4:**
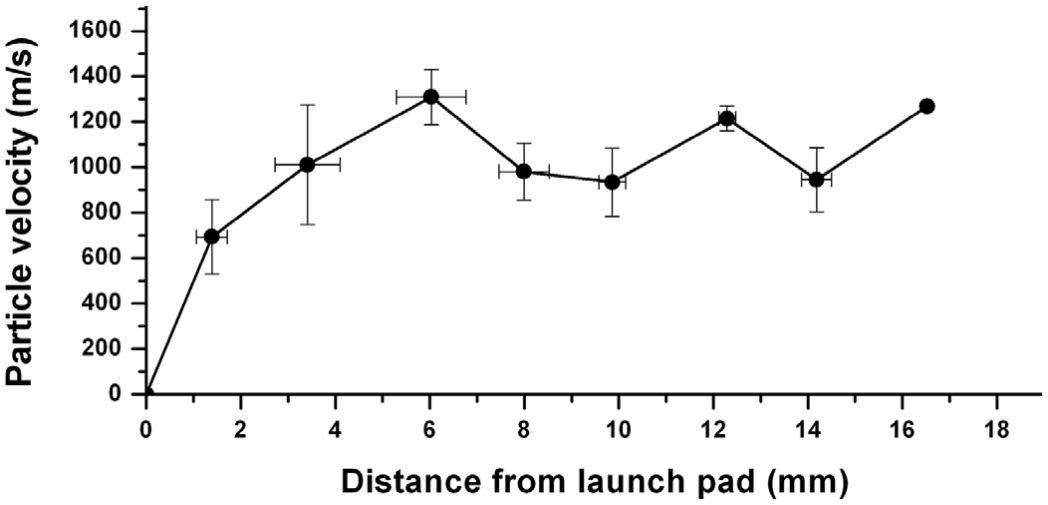
Velocity of the microparticles with respect to distance from the launch pad, deduced from the visualized pictures. Scatter bars indicate standard deviation.

The average velocity of the tip of the particle cloud, measured from the visualized photographs is plotted in Fig. 4, with respect to the distance from the launch pad. The particle cloud accelerated on leaving the launch pad over a distance of about 5 mm, then attained a quasi-steady, average velocity of 1100 m/s for a distance of 10 mm, approximately, before declining. The acceleration and maintenance of a high velocity by the microparticles for a substantial period is the main advantage of the present mode of operation of the device in comparison with its previous version [9], where the particles decelerated quickly on leaving the launch pad, limiting the use to soft targets.

**Figure 5:**
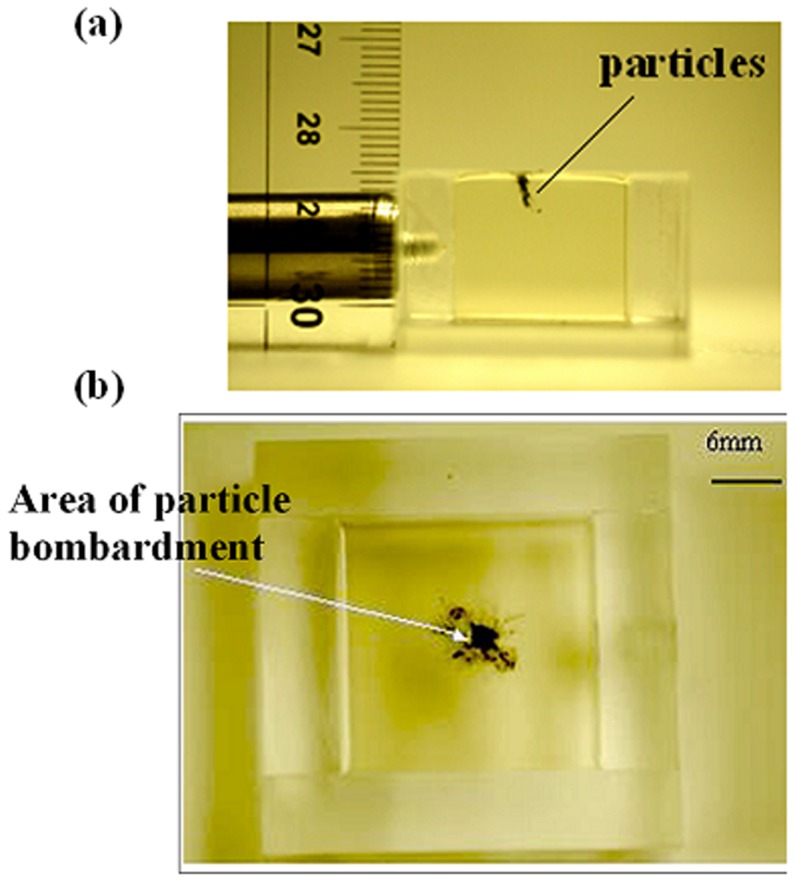
Delivery of microparticles into a soft *in vitro* target. (a) Penetration of 1-µm size tungsten particles into 3% gelatin; target standoff distance: 3 mm. 500 µg of tungsten particles were delivered. (b) The top-view indicating the territory of particle bombardment.

The particle delivery was initially tested on a gelatin slab of 3% concentration to analyze the impact on the target. Tungsten particles of 1 µm size were used on this *in vitro* target, which was placed at a standoff of 3 mm from the launch pad. Tungsten particles were suspended in 70% ethanol at a concentration of 100 mg/ml, and 5 µL of this suspension was deposited on the aluminum foil prior to delivery, which quantified to 500 µg of particles. The 3% gelatin (strength: 20–25 bloom; cooled at 10°C for 1 hour) represents human thrombus [10], and its percentage was determined by the weight ratio of gelatin to water. The penetration of particles through gelatin is shown in Fig. 5. The top view of the gelatin model in Fig. 5b shows the spread of the particles on the target surface on bombardment. The effective area of bombardment in which the particles penetrated the target was observed to be about 6 mm^2^ (±1 mm^2^) in gelatin. The effective area was expected to change (slightly) with the target standoff from the launch pad, as the particle cloud diverged under the influence of plasma jet.

**Figure 6:**
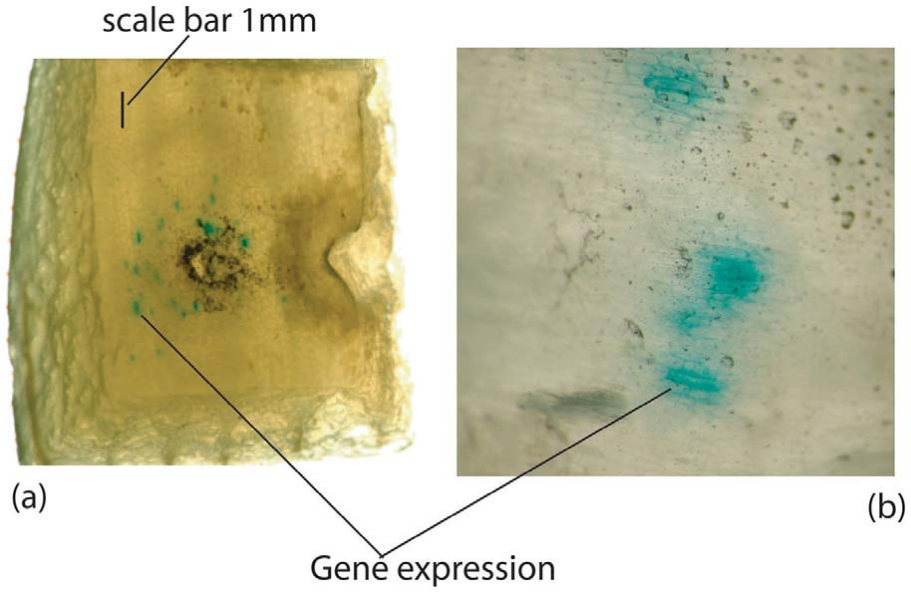
Onion scale cells showing GUS (gene) expression. (a) A macrograph indicating the area of particle bombardment and the extent of gene expression. (b) A micrograph highlighting the gene expression spots.

The *in vivo* results of DNA delivery into scales of onion are presented in Fig. 6. DNA coated gold microparticles of 1 µm size were suspended in 70% ethanol at a concentration of 60 mg/ml, and 5 µL of the suspension was deposited on the aluminum foil prior to delivery into the *in vivo* targets, which amounted to 300 µg of particles, containing 4.5 µg of the plasmid DNA. The blue spots in the onion cells indicate the GUS activity in the cells. Several tests were carried out to ensure the repeatability of the DNA delivery. No blue spots were detected on bombarding the targets with uncoated gold particles. The coated DNA was effective and uncontaminated during the process of delivery, though the coated particles were carried onto the target by a plasma jet. The biological target (onion) was placed at a standoff distance of 10 mm from the launch pad/foil. The particle-spread was over an area of about 9 mm^2^ (±1 mm^2^ standard deviation) on the onion scales, and about 80 cells (±10 standard deviation) were transfected per delivery. The average number of onion cells in an area of 9 mm^2^ was about 225, of which about 80 cells expressed GUS activity on being bombarded by the DNA particles.

The size of onion cells is quite large in comparison with mammalian [11], lamprey brain [4] and plant cells such as soybean seed and tobacco leaf [8], due to which the number of cells in a given area of particle bombardment was less comparatively. But the onion scales represent harder living targets that the device practically aims at, and a transfection of 80 cells on an average per delivery was quite comparable to the transfection achieved using Helios Gene Gun on lamprey brain cells, which was of the order of 100 cells for neurons and 200 cells for glia, on an average. The lamprey neuron and glial cells were approximately of the size of 20 µm and 10 µm, respectively [4], and the glial cells had a better scope of transfection owing to their higher density in the territory of bombardment due to smaller cell size. The transfection efficiency observed on mammalian cells using Helios Gene Gun was 42.8% [11], which was on par with the transfection efficiency of 35.6% (80/225) observed in the onion scale cells in the present case, considering higher density of mammalian cells in a given area.

The present device primarily propels the drug particles due to inertia, which are further aided by the plasma jet on their path to the target. The plasma in the device is generated by the ablation of a thin aluminum foil by a laser pulse of 5.5 ns duration, which cools immediately after release from the device due to sudden expansion to atmospheric pressure (1 bar). The dissemination of the coated DNA in the living target and the subsequent gene expression by the living cells bear testimony to the fact that the deployed plasma jet is quite a safe and an effective carrier for the drug particles. While the device has several advantages such as miniaturization to adapt to minimally invasive surgical devices, minimal intrusion of the carrier gas and the device accessories (spacers), extremely good controllability being laser driven, etc., which are apt for non-approachable, internal, hard targets in human body, the outlay of the device works out to be slightly higher than the biolistic devices cited above. The cost is primarily due to the laser, but the functional attributes of the device are likely to suppress the cost factor. The other docile drawback of the device includes the minute foil particles on rupture accompanying the drug particles into the target. The rupture of the foil can be modulated by inscribing minute V-grooves in its surface, which can guide the foil rupture thereby minimizing the generation of foil particles during the operation. However, use of V-grooved, biocompatible launch pads made of metals such as gold, silver or titanium in actual clinical operations will subdue the problem associated with the launch-pad-particles. The material of the biocompatible launch pad can be chosen based on the medical application. The grooved biocompatible launch pads will be used in the future phase of our research.

### Conclusion

A laser-based particle delivery device to deliver drug-coated microparticles into biological targets has been tested. The device physics has been investigated through a high-speed photography of the particle acceleration process. The device has been tested for DNA delivery on living scales of onion. The preliminary, *in vivo* experimental results show that it is possible to have an effective drug delivery using this method.
